# Unmasking the Silent Invader: A Rare Case of Follicular Thyroid Carcinoma With Skull Metastasis and an Uncommon KRAS Q61R Mutation

**DOI:** 10.7759/cureus.47641

**Published:** 2023-10-25

**Authors:** Hehua (Hannah) Huang, Ping Ji, Shi-Kaung Peng

**Affiliations:** 1 Pathology and Laboratory Medicine, Harbor University of California Los Angeles Medical Center, Torrance, USA

**Keywords:** targeted treatments, molecular characterization, ihc staining, ras mutation, kras q61r mutation, next-generation sequencing (ngs), thyroid ultrasound, t3 hyperthyroidism, skull metastasis, follicular thyroid carcinoma (ftc)

## Abstract

Follicular thyroid carcinoma (FTC) is a noteworthy subtype of thyroid cancer known for its tendency to metastasize through the bloodstream, usually to the lungs and bones. This case report examines an exceptionally rare instance involving an 81-year-old female presenting with an unusual metastatic scalp lesion. Remarkably, this aggressive metastasis originated from a thyroid lesion as small as 0.7 cm. Lab findings, including suppressed TSH and elevated T3 levels, revealed subclinical hyperthyroidism, adding another layer of rarity to this FTC case. Molecular profiling identified a rare KRAS Q61R mutation, providing potential insight into the case's aggressive behavior and underscoring the importance of genetic assessment in FTC. This report emphasizes the critical role of comprehensive diagnostic evaluations, including histopathological assessments, in properly diagnosing and managing FTC, especially when clinical presentations defy conventional paradigms.

## Introduction

Thyroid carcinoma stands as the most common malignancy within endocrine system cancer, accounting for nearly 1% of malignancies [1]. Within this framework, follicular thyroid carcinoma (FTC) is the second most prevalent after papillary thyroid carcinoma (PTC), representing about 5% to 15% of all thyroid malignancies [2,3]. Demographically, FTC typically emerges in individuals between 40 and 60 years of age and exhibits a higher prevalence in females [2].

Differentiating between the types of thyroid carcinomas reveals distinct patterns of metastatic behavior. FTC is characterized by its predisposition for distant metastasis, especially to the lungs and bones [2]. In contrast, PTC frequently manifests with lymph node metastases. While roughly 30% of FTC cases experience distant metastasis to the lungs or bones, this is seen in only 15% of PTC cases [2]. Among these, metastases from thyroid carcinomas more commonly target the spine [4]. Exceptionally rare is the metastatic progression of FTC to the skull, which occurs in less than 1% of FTC cases [4,5]. The limited documentation of such instances in the medical literature, mostly presented as individual case reports [5-10], emphasizes their rarity.

## Case presentation

Clinical background

An 81-year-old female with a history of hypertension and asthma presented with a large growing scalp mass on the right posterior aspect of her head. Initially 1 inch in diameter, the mass was excised in Mexico due to a suspected lipoma, which resulted in massive bleeding and necessitated a hospital transfer. However, no pathology diagnosis was available, and the mass recurred and grew over the past year. The patient reported no symptoms of hyperthyroidism or hypothyroidism and had no palpable thyroid masses. She also experienced recurrent left-sided epistaxis.

Imaging studies

**Figure 1:**
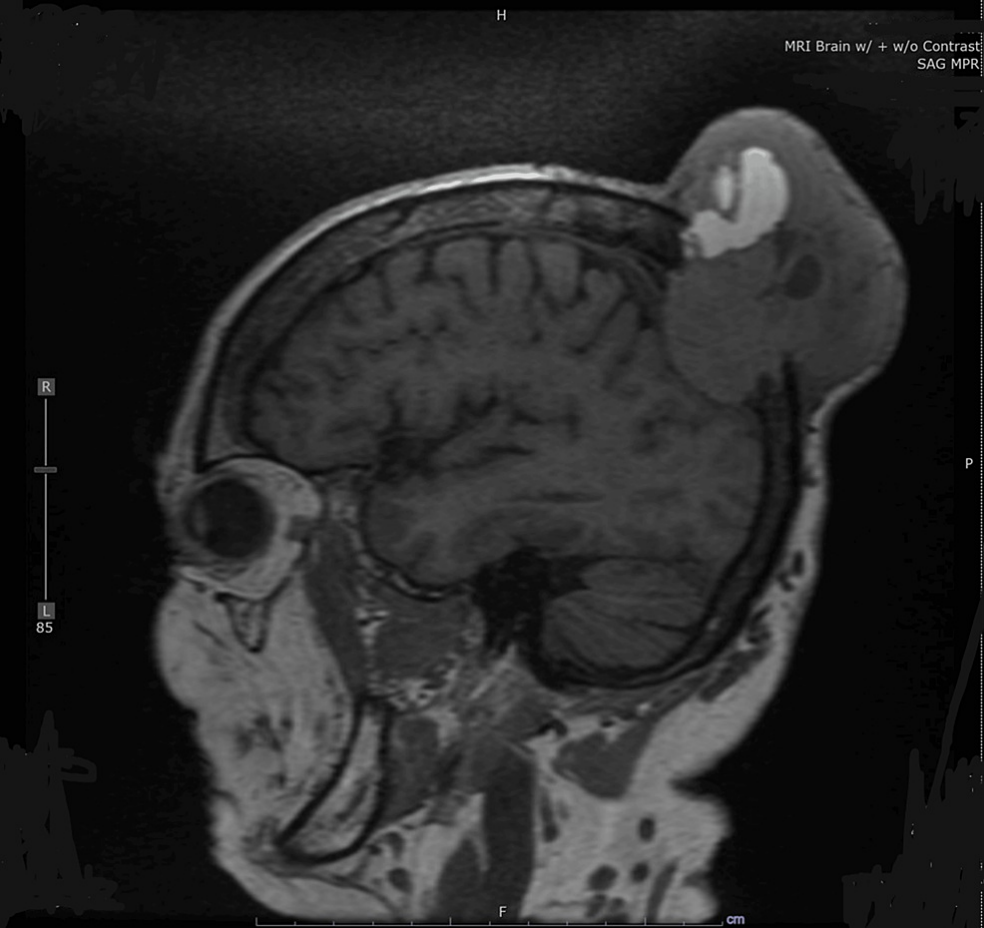
Sagittal MRI of the head with and without contrast. MRI of the brain depicting a large 8.9 cm x 9.2 cm x 7.4 cm solid-cystic mass in the posterior parietal calvarium with bony destruction and both extracranial and intracranial extensions. MRI, magnetic resonance imaging

**Figure 2:**
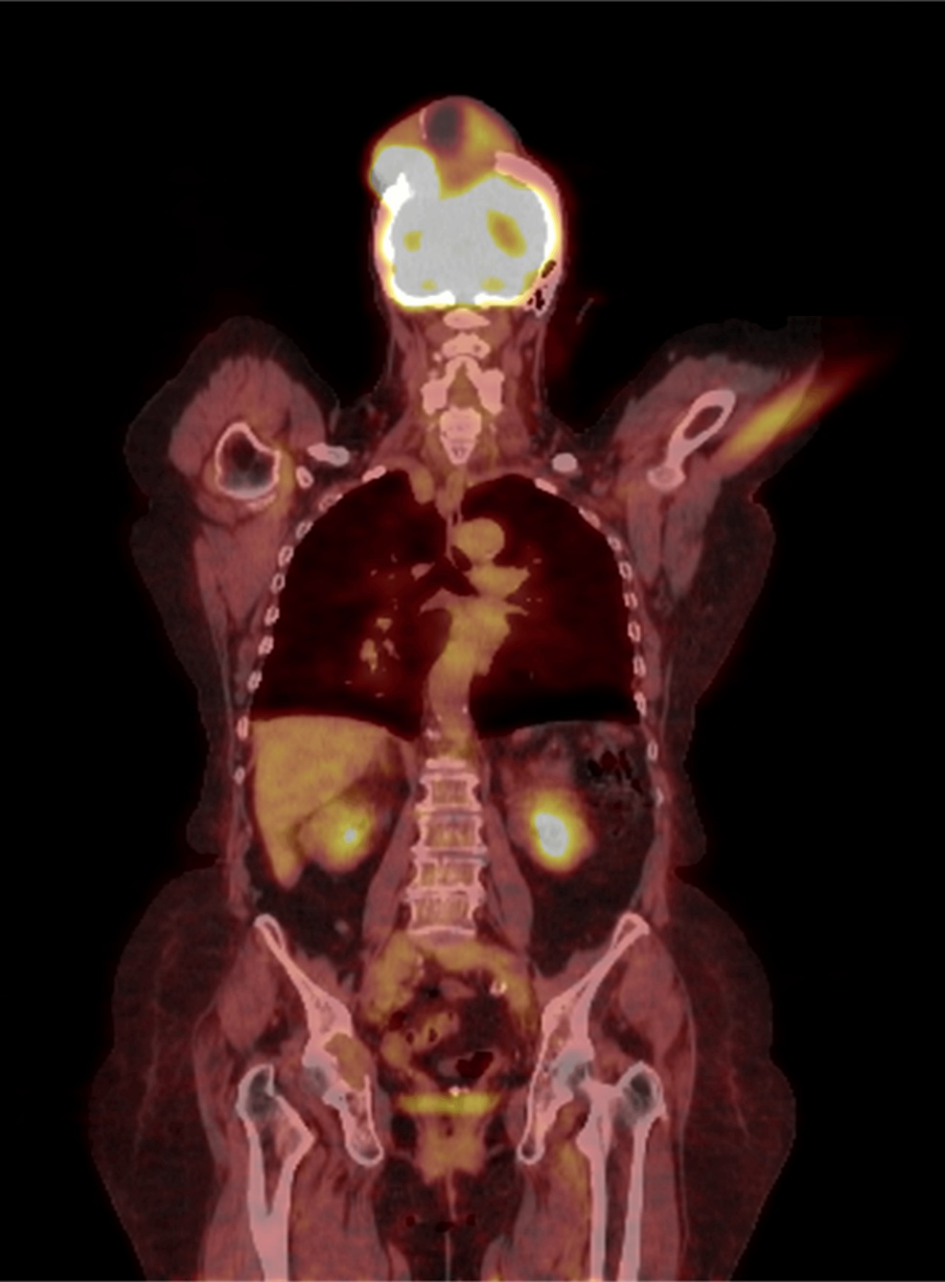
Whole-body PET/CT scan. The PET/CT scan revealing a calvarial mass with significant bony destruction and additional calvarial lesions. PET/CT, positron emission tomography/computed tomography

A comprehensive evaluation was performed using a computed tomography (CT) scan, magnetic resonance imaging (MRI; Figure 1), and positron emission tomography (PET)/CT scan (Figure 2). The CT scan delineated two hepatic hypervascular lesions and multiple bone lesions indicative of metastatic disease. Additionally, bibasilar pulmonary nodules and a substantial vascular mass with calvarial destruction were noted in the right posterior vertex, encompassing both extracranial and intracranial components. The MRI of the brain unveiled a substantial 8.9 cm x 9.2 cm x 7.4 cm solid-cystic mass emerging from the posterior parietal calvarium. This mass was characterized by destructive bony changes, extracranial extension into scalp soft tissues, and intracranial extension suggestive of probable dural invasion. In the PET/CT scan, an irregular mass displaying extensive bony destruction was observed, with both extracranial and intracranial manifestations similar to those noted on MRI. Additional calvarial lesions were also identified.

Histopathology

**Figure 3:**
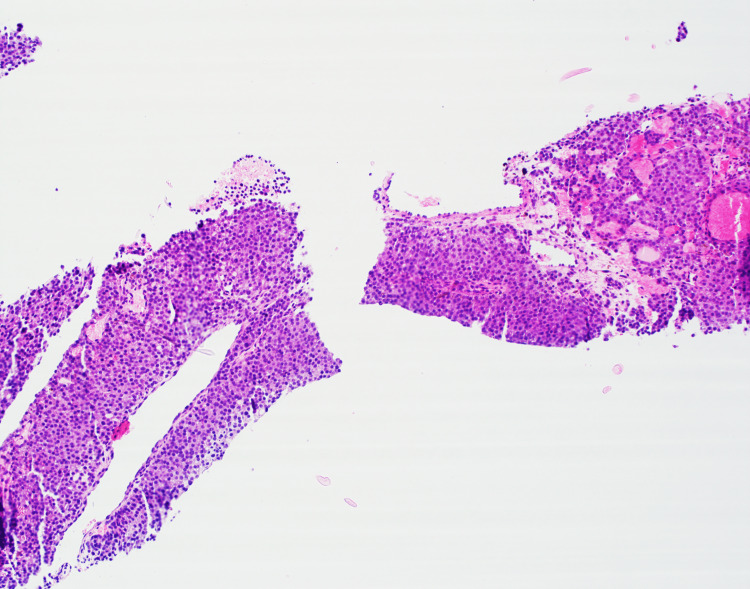
Core biopsy of the parietal lesion, Hematoxylin and Eosin stain, 40x magnification. Sheets of small, round, uniform cells are intermixed with abundant capillaries and vessels. The formation of follicular and microfollicular architecture, along with the faint presence of pink-red colloid-like secretions in the lumen, hints at a thyroidal origin.

**Figure 4:**
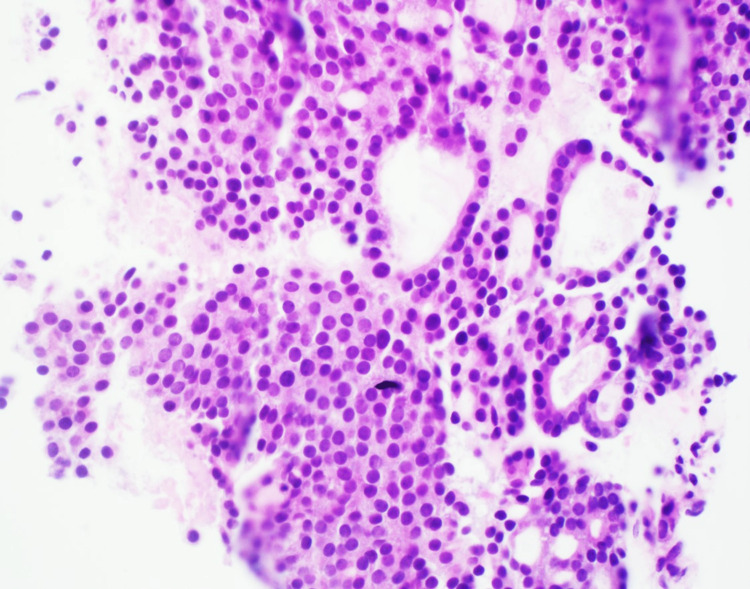
Core biopsy of the parietal lesion, Hematoxylin and Eosin stain, 400x magnification. The histopathological view displays cohesive, small, round, uniform cells organized into follicular and microfollicular patterns. The subtle pink-red colloid-like secretions within the lumen are suggestive of thyroid adenocarcinoma of the follicular type.

The histopathological evaluation of the core biopsy from the pronounced parietal scalp lesion (Figures 3-4) revealed sheets of cohesive, small, round, uniform cells intermingled with abundant capillaries and vessels. Their formation into follicular and microfollicular patterns, combined with the subtle pink-red colloid-like secretions in the lumen, suggested thyroid adenocarcinoma of the follicular type. The absence of hallmark features, such as nuclear grooves or inclusions typically seen in PTC, steered the differential diagnosis away from this common form of thyroid cancer, especially its follicular variant. The subtle presence of colloids and the formation of a few glandular structures raised the possibility of metastatic adenocarcinoma originating from the lung or other organs.

**Figure 5:**
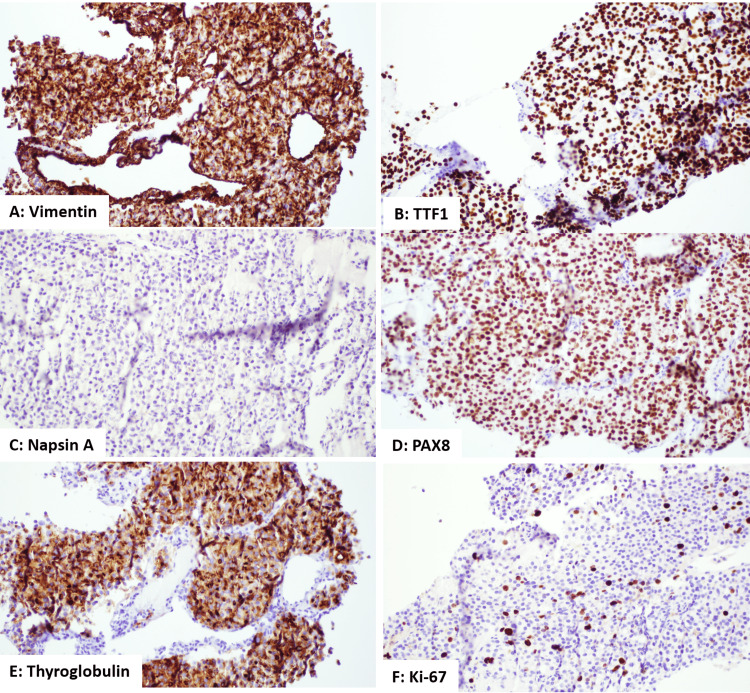
Immunohistochemistry (IHC) stain of the parietal lesion core biopsy: (A) Vimentin+, (B) TTF1+, (C) Napsin A-, (D) PAX8+, (E) thyroglobulin+, (F) Ki-67 (10%-20%).

From the microscope results, it seemed the diagnosis was a follicular-patterned lesion that might be an adenocarcinoma. However, the main origin was not clear. We did more tests using immunohistochemical stains. First, we looked at markers AE1/AE3, Cam5.2, CK7, Vimentin (Figure 5A), TTF1 (Figure 5B), and Napsin A (Figure 5C). The tumor cells tested positive for AE1/AE3, Cam5.2, CK7, Vimentin, and TTF1. This suggests it could be from the thyroid. However, since there was no positive result for Napsin A, it's less likely to be a primary lung adenocarcinoma.

To further confirm the thyroidal origin, we conducted additional IHC tests targeting thyroglobulin and PAX8. The tumor cells exhibit strong positivity for PAX8 (Figure 5D) and thyroglobulin (Figure 5E). When considered alongside the earlier positive findings for CK7 and TTF1, this confluence of results firmly pointed toward a diagnosis of thyroid follicular adenocarcinoma. Additionally, a heightened Ki-67 proliferation index of 10% to 20% (Figure 5F), underscored the aggressive growth potential observed in the tumor cells of this case.

However, a holistic diagnosis also considers markers that the tumor cells do not express. The absence of P63 and CK5/6 made squamous cell carcinoma an unlikely diagnosis. Synaptophysin's nonexpression eliminated the possibility of a neuroendocrine tumor. The tumor's negative markers for CK20 and EMA diminished the likelihood of gastrointestinal, as well as a myriad of other carcinomas. Additionally, WT1 and CDX2's absence further indicated the tumor was not of genitourinary or gastrointestinal tract origin. Importantly, collective negative staining for Gata3, Mammaglobin, ER, PR, and BRST2 firmly excluded a breast primary.

Through this detailed and methodical approach, using both the presence and absence of markers, the diagnosis was refined. The aggregate evidence pointed toward FTC, emphasizing the invaluable role of comprehensive histopathological and IHC assessment in intricate cases.

Further lab tests, imaging, and molecular studies

Thyroid function tests revealed a suppressed TSH level of less than 0.010 microIU/mL, a slightly elevated free T3 level of 4.7 pg/mL, and a normal T4 level of 1.00 ng/dL. This unique hormone profile indicated T3 hyperthyroidism - an unexpected lab finding considering the patient's lack of clinical symptoms.

Following these results, a comprehensive thyroid ultrasound pinpointed a Reporting and Data System (TI-RADS) category 5 (TR5) lesion within the left thyroid lobe, measuring up to 0.7 cm. This lesion was solid and displayed deep hypoechoic attributes. Although other thyroid anomalies emerged, they did not conform to the American College of Radiology (ACR) thyroid imaging criteria demanding invasive assessment or ongoing monitoring.

To explore the molecular mechanisms behind this case, we conducted comprehensive next-generation sequencing (NGS) on the patient's scalp mass biopsy specimen in formalin-fixed paraffin-embedded (FFPE) tissue. This involved meticulous analysis of both DNA and RNA through targeted NGS, to identify mutations, evaluate copy number variants, and detect gene fusions. The rigorous NGS analysis revealed a crucial discovery: a Kristen RAt Sarcoma viral oncogene homolog (KRAS) Q61R mutation (c.182A>G) present in a significant 90% of neoplastic nuclei. This mutation, while known to be associated with various cancers, held particular importance in our case. Its presence may have played a contributory role in the aggressive metastatic presentation observed, a stark contrast to the surprisingly quiescent clinical manifestations.

Despite our comprehensive diagnostic insights, the patient expressed reluctance toward undergoing any medical procedures, surgery, or radiation therapy. At the time of our last contact, she was in Mexico and was subsequently lost to follow-up. This scenario underscores the complexity and unpredictability of thyroid malignancies and the importance of consistent patient monitoring and engagement.

## Discussion

Our case sheds light on an exceptionally rare presentation of thyroid carcinoma, standing out as a noteworthy deviation from conventional clinical scenarios. Our patient, while not exhibiting overt hyperthyroid symptoms, displayed laboratory findings consistent with subclinical hyperthyroidism, including suppressed TSH, elevated T3, and normal T4 levels. It's notably rare for FTC to manifest alongside hyperthyroidism [11,12].

The rare presentation of hyperthyroidism in our patient can be attributed to either an autonomous functioning thyroid nodule or from functioning metastases (FM) [12]. Two predominant mechanisms are believed to underlie the development of FM. First, activating mutations in the thyroid-stimulating hormone receptors (TSHRs) or the stimulatory guanine nucleotide-binding protein subunit alpha s (Gsa) can lead to perpetual activation of the cyclic adenosine monophosphate-protein kinase A (cAMP-PKA) pathway, inducing hyperthyroidism. Second, tumor cells might overexpress 5′-iodothyronine deiodinase, thereby enhancing the conversion of levothyroxine to T3 [12]. This uncommon hormonal manifestation in FTC underscores the distinctive nature of our patient's case.

This case underscores the pivotal role of meticulous histopathological evaluations in unraveling complex clinical presentations. While the observed follicular architecture provided a crucial clue, the case's atypical clinical history and manifestation serve as reminders that thyroid carcinomas can adeptly mimic other malignancies, even when the primary lesion remains silent.

The systematic use of immunohistochemical staining was important in this diagnosis. It was not merely the presence of certain markers, like TTF1 and CK7, but also the absence of others that steered the diagnosis toward FTC. Each negative stain played its part in whittling down the list of potential primary sites.

Importantly, the combination of CK7, TTF1, PAX8, and thyroglobulin confirmed the thyroidal origin, emphasizing the nuanced capabilities of modern histopathology. Such refined diagnostic tools are instrumental in ensuring patients receive the most appropriate care based on their unique pathology.

The imaging findings offered a compelling juxtaposition: a diminutive 0.7 cm lesion in the left thyroid gland contrasting sharply with the aggressively expansive metastatic scalp mass. This stark contrast accentuates the unpredictable behavior of certain thyroid tumors, underscoring the fact that size is not always indicative of metastatic potential or aggressiveness. In this patient's case, the absence of overt hyperthyroid symptoms, perhaps muted by her advanced age, coupled with the small primary lesion size, might have led to an initial underestimation of the tumor's potential reach. This case serves as a stark reminder that even subtle primary thyroid lesions can be deceptive and warrant comprehensive evaluation and follow-up.

The RAS gene family, encompassing KRAS, Neuroblastoma RAt Sarcoma viral oncogene homolog (NRAS), and Harvey RAt Sarcoma viral oncogene homolog (HRAS), stands as a cornerstone of mutational events in a diverse array of cancers. Notably, KRAS mutations are prevalent in cancers such as non-small cell lung cancer (NSCLC), colorectal cancer, and pancreatic cancer, while NRAS and HRAS mutations are often delineated in melanoma, leukemia, and certain thyroid cancers [13,14].

FTC particularly displays genetic alterations like RAS point mutations and Peroxisome Proliferator-Activated Receptor Gamma (PPARG) rearrangements, with NRAS mutations, especially at codon 61, being predominant [2,15-18]. Recent explorations into this phenomenon, conducted across a spectrum of thyroid tumor subtypes, unveil KRAS mutations in a critical 6% of FTC cases, with a striking 57% of mutations distinctly positioned at codon 61. The exclusive identification of KRAS and TP53 mutations within borderline or manifestly malignant tumors underscores their prospective contribution to tumor virulence and malignancy [19].

Specific hotspots within KRAS (codon 12, 13, 61, or 146) harbor mutations that can significantly hyperactivate downstream effector pathways like the Mitogen-Activated Protein Kinase (MAPK) and Phosphoinositide 3-Kinase-Protein Kinase B (PI3K-AKT) signaling cascades. The Q61R mutation exemplifies such hotspot mutations [20]. Upon activation, KRAS orchestrates a series of signaling pathways, including the RAF-MEK-ERK (MAPK) and the PI3K-AKT-mTOR pathways, pivotal for cell proliferation, survival, and other cellular functionalities. The dysregulation of these pathways due to KRAS mutations potentially accelerates cancer progression [2,21].

Historically tagged as *undruggable*, KRAS has transitioned into the limelight with the genesis of specific inhibitors, prominently against the G12C mutation. For instance, AMG510 (Sotorasib) irreversibly binds to Cys12, rendering KRAS(G12C) inactive, and received U.S. Food and Drug Administration (FDA) approval on May 28, 2021, marking a pivotal milestone in KRAS-targeted therapy [21].

Many clinical trials are underway, probing novel drug combinations like the fibroblast growth factor receptor (FGFR) Inhibitor Futibatinib coupled with MEK-inhibitor Binimetinib (ClinicalTrials.gov ID NCT04965818), or evaluating novel agents like BDTX-4933 in the treatment of BRAF and KRAS (beyond G12C variants like G12D and G12V) advanced neoplasms, including thyroid cancer (ClinicalTrials.gov ID NCT05786924). Yet, a pronounced gap remains in targeted interventions for the KRAS Q61R mutation, highlighting a crucial realm for future investigative endeavors.

While targeted therapies for KRAS Q61R mutations continue to be scant, insights have been unearthed through studies identifying the concordance of KRAS Q61K and silent mutations G60G/A59A. This understanding has enabled the exploration of thwarting exonic splicing enhancer (ESE)-mediated splicing with mutant-specific oligonucleotides, thereby rendering the RAS(Q61) protein nonfunctional. This approach has showcased potential therapeutic effects both in vitro and in vivo, heralding a potential therapeutic strategy for future KRAS Q61-targeted interventions [22].

Despite the advancements, challenges persist, encompassing the understanding and surmounting of both intrinsic and acquired resistances to KRAS inhibitors [2,21,22].

## Conclusions

In this compelling case, an 81-year-old female presented with a significant metastatic scalp mass, notably linked to a minor 0.7 cm thyroid lesion. The diminutive nature of the primary tumor starkly contrasted with its aggressive metastatic prowess, underscoring the unpredictable behavior inherent to FTC. Despite its silent presentation, lab tests unmasked an unexpected subclinical hyperthyroidism, evidenced by suppressed TSH and slightly elevated T3, an anomaly seldom associated with FTC.

Further molecular analysis unveiled the presence of the less common KRAS Q61R mutation, often implicated in various malignancies. This mutation might offer a clue to the tumor's aggressive disposition in our patient. Given the critical role of KRAS mutations in regulating cell growth and survival, they pave the way for potential therapeutic interventions, with agents like Sotorasib and BDTX-4933 demonstrating preliminary promise. This case serves as a poignant reminder of FTC's potential to mimic other malignancies, emphasizing the importance of a comprehensive and collaborative approach in its diagnosis and management, especially when clinical presentations challenge conventional norms.
